# Parents’ Perceptions and Experiences with Their Children’s Use of Augmentative/Alternative Communication: A Systematic Review and Qualitative Meta-Synthesis

**DOI:** 10.3390/ijerph19138091

**Published:** 2022-07-01

**Authors:** Carmen Berenguer, Eva Rosa Martínez, Simona De Stasio, Inmaculada Baixauli

**Affiliations:** 1 Department of Developmental and Educational Psychology and ERI-Lectura, University of Valencia, Avda. Blasco Ibáñez, 21, 46010 Valencia, Spain; 2 Department of Basic Psychology and ERI-Lectura, University of Valencia, Avda. Blasco Ibáñez, 21, 46010 Valencia, Spain; eva.rosa@uv.es; 3 Department of Human Studies, LUMSA University, 00193 Rome, Italy; s.destasio@lumsa.it; 4 Campus Capacitas, Catholic University of Valencia, 46010 Valencia, Spain; inmaculada.baixauli@ucv.es

**Keywords:** metasynthesis, thematic analysis, parents’ perspectives, augmentative and/or alternative communication, qualitative

## Abstract

Augmentative and alternative communication (AAC) consists of any method of communicating that supplements or completely substitutes oral and/or written language when it is impaired. Therefore, it enables children with complex communication needs to develop their full communicative potential. However, despite the many benefits of AAC and its widespread use, several review studies have underscored the problems faced by parents and children who use AAC in their daily lives. The general objective of this systematic review and qualitative meta-synthesis is to provide a complete overview of parents’ experiences and perceptions with their children’s use of AAC. Specifically, it aimed to identify common themes and subthemes of interest and to analyze the research quality of the selected studies. An exhaustive literature search was carried out using different electronic databases. Nineteen studies were included, involving 297 parents. A thematic synthesis was undertaken. Three main themes and nine subthemes were identified: service support (accessibility, providers and coordination); characteristics of AAC systems (usability and acceptability, features, cost and funding); and integration of AAC in daily life (family, school, social and community). Findings raise a need for more services that support children with complex communication deficits in different contexts, more functional use of AAC systems at school and in real-world situations, as well as service assistance over an extended time period.

## 1. Introduction

Communication skills are essential in the adaptive development of individuals, especially during childhood, and are often considered a major factor that positively influences short- and long-term outcomes [1]. In consequence, children and adolescents with receptive and expressive communication deficits experience challenges across many life areas. A lot of studies have reported that they show higher rates of psychosocial impairments, lower academic performance, more peer rejection, and difficulties in their social interactions, compared to typically developing peers [2].

People with complex communication needs are a heterogeneous group including individuals with a wide range of disabilities as Autism Spectrum Disorder, intellectual disability, communication disorders, cerebral palsy, Fragile X, Angelman syndrome, Down syndrome, Rett syndrome or others.

The prevalence of individuals with communication and language problems is increasing due in part to the growing incidence of ASD [3]. Moreover, some studies have reported that approximately 8% of children between the ages of 3 and 17 years in the United States experience language and communication difficulties, and therefore may require additional support to supplement their communication skills [4]. In this sense, augmentative and/or alternative communication (AAC) can help to improve some of the challenges faced by people with communication disorders [5].

AAC is an umbrella term that encompasses all the methods and means of communication intended for helping/replacing speaking and/or writing when these are being affected. It includes a wide range of unaided and aided strategies and low or high techniques to assist the communication and participation of individuals with complex communication needs. Unaided systems refer to manual signs and gestures to support the communicative interaction. Aided communication modes require additional materials or devices outside of the speaker’s body and are subdivided into high- and low-technology. Low-technology systems or devices encompass communication books or boards (non-battery powered), written words on paper, line drawings, symbols on a ring or in a notebook, photographs, and pictograms such as the Picture Exchange Communication System (PECS; Bondy and Frost [6]). High-technology systems include speech generating devices (SGD) or voice output communication aids (VOCAs), and software on tablets, computers, mobiles, iPads, and/or apps used as communication aid [7,8]. Moreover, aided AAC systems are most often selected for individuals with complex communication needs and can contribute to more positive outcomes for this population [9].

Despite the enormous potential of AAC and the exponential increase in aided high technology systems, several review studies have highlighted the problems faced by parents and children who use AAC in their daily lives. As a result, some families with children and adolescents with complex communication needs end up abandoning or under-using AAC systems. A study by Moorcroft et al. [10] evidenced that only 39.35% of AAC systems were used for more than one year in a survey conducted among speech-language pathologists (SLPs).

There is little research that has synthesized parental experiences with augmentative and alternative communication. Moorcroft et al. [11] identified 43 studies to provide a synthesis of the barriers and facilitators about the use of low-tech and unaided AAC systems from the perspective of people of a wide range of age and diseases with complex communication needs, their families, and the professionals involved. Although the study presents some methodological limitations, the results showed the need of training in the use of AAC systems by some professionals, including SLPs and teachers and the organizational barriers to the implementation of AAC. The authors concluded that the provision and use of AAC systems is influenced by several personal factors such as parents’ acceptance of their children disability, their success in using AAC, or the support provided by other parents in similar circumstances. Another review focused on examining the effectiveness of AAC interventions for children with Autism Spectrum Disorder (ASD) and identified four potential mediators of intervention outcomes: communication partner knowledge; perception of benefits of the AAC system used; adult-family input at home, and the frequency of AAC exposure provided during therapy sessions [12]. Furthermore, a review devoted to analyzing the barriers and facilitators to the use of AAC systems by children with ASD and their communication partners, evidenced the variability of outcomes across individuals, AAC modalities, and environments. Results suggested that service providers had different levels of knowledge and understanding of evidence-based practice of AAC systems and SLPs should identify barriers and facilitators of using AAC to support families to make informed intervention decisions [13]. Similarly, White et al. [14] evaluated the effects of AAC on speech development in children with ASD, highlighting that it is still unclear if the addition of AAC will benefit a participant’s speech production. More recently, a mega-review summarized published peer-reviewed literature reviews about aided AAC interventions that included children with developmental disabilities [8]. The need for increasing generalization and maintenance programming and ensuring that lasting and social important behaviors occur with the use of AAC was highlighted. 

Examination of the facilitators and barriers that may exist for parents to the provision and children’s use of AAC systems could highlight some important considerations for the effectiveness, accessibility, and maintenance of AAC systems. However, previous reviews have been limited by methodological constraints or for not considering parents’ perceptions about their children’s use of AAC. Qualitatively exploring the perceptions and experiences of parents about AAC has the potential to identify the key factors that contribute to supporting children with complex communication needs and their families. Therefore, the current review examines the perceptions and experiences of parents with their children’s use of AAC systems.

Specifically, the present systematic review and meta-synthesis tries to respond to the following objectives and research questions:

(a) To provide a complete overview of parents’ experiences and perceptions regarding the use of AAC by their children with complex communication needs in order to identify, integrate, and interpret common themes which may contribute to improving professional practice:
  - What do families think about the support they receive during the process of AAC implementation?
  - What are the characteristics of the AAC that constitute barriers and facilitations to their use (accessibility, portability, design, economic cost, etc.)? What are the reasons that lead some families to abandon the use of AAC?
  - What challenges are posed by the different contexts of use of the AAC?
(b) To find out the quality of the investigations about family’s experiences and opinions on the use of AAC by their children, with the aim of determining the rigor of the research base to date, and the aspects needed to be overcome in future studies.

The findings of this qualitative metasynthesis may be used to help identify ways to improve the implementation of AAC to optimize language and communication skills for children with complex communication needs. It has important implications for stakeholders (parents, SLPs, teachers, medical services, community) in understanding AAC systems and providing support to children with communication difficulties and their families. 

## 2. Methodology

### 2.1. Search Strategy and Screening Process

A systematic literature search was undertaken from November 2021 to December 2021 using the following electronic databases: Pubmed, CINAHL, PsyINFO, Embase, Web of Science Core Collection, Education Resources Information Centre (ERIC), Scopus, gray literature databases (digital thesis), and generic web searches (Google Scholar). The identified studies were included using the SPIDER tool (Sample, Phenomenon of Interest, Design, Evaluation, Research Type) [15]. Therefore, the searches were carried out using Boolean operators AND/OR by the following combination of these terms in either the title, abstract or keywords or “topic” depending on the settings allowed in each database: S-sample (parent* OR mother* OR father* OR caregiver*); PI-phenomenon of interest (augmentative and alternative communication OR AAC OR communication device OR aided communication OR aided language OR picture exchange OR sign language); D-design (perception* OR perspective* OR opinion* OR experience* OR belief* OR view* OR attitude*); E-evaluation (interview* OR focus group* OR questionnaire* OR survey*) and R-research type (qualitative OR mixed method).

**Figure 1:**
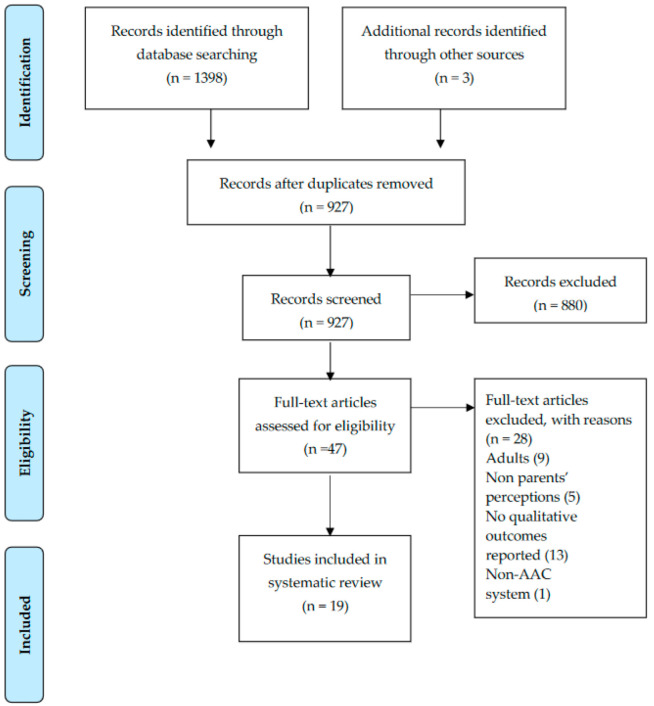
PRISMA flowchart of study selection process.

This review was conducted in accordance with the Preferred Reporting Items for Systematic Reviews and Meta-Analyses (PRISMA) guidelines [16], see Figure 1.

The first and second authors independently searched the literature and screened all titles and abstracts. Agreement between reviewers after screening the potential studies was high (kappa = 0.95). If a full text met all the predefined eligibility criteria, it was included in the review. Any uncertainty on meeting the eligibility criteria was resolved by consensus among the research team.

### 2.2. Exclusion and Inclusion Criteria

Papers were included if they: (i) were written in English, (ii) used qualitative methods and/or mixed methods, as long as it was possible to extract the qualitative data collection, narrative analysis (specifically interviews or focus groups, surveys), (iii) involved parents or caregivers of children (aged 2–17 years) users of augmentative and/or alternative communication systems, with any type of speech, language, or communication difficulty, (iv) focused on parents’ views, experiences or perceptions of the use of augmentative and/or alternative communication, (v) were published in a peer-reviewed journal, and (vi) were published from 2012 to January 2022. This time frame was selected following Moorcroft [11], as in the last decade, there have been increasing innovations in the development of high-tech AAC systems and little change in unassisted, low-tech AAC systems and this is important to consider when analyzing parents’ perceptions of their children’s use of AAC systems.

Papers were excluded if: (i) studies did not include the experiences and perceptions of parents, (ii) had a literacy focus (i.e., reading, reading comprehension, and writing), (iii) did not report qualitative outcomes, (iv) did not employ AAC systems, or (v) parents or caregivers of adults were involved.

Considering the large number definitions of AAC offered in the literature, we adopted the definition by Crowe et al. [8], for a study to be included in the current review. Therefore, AAC comprises unaided systems such as manual signs and gestures as well as aided systems such as the Picture Exchange Communication System and speech generating devices (SGD).

### 2.3. Methodological Quality Evaluation

The quality of included studies in this review was appraised using the Program Qualitative Checklist (Critical Appraisal Skills Program (CASP), 2018), a strong quality assessment tool for assessing qualitative research. The ten items on the CASP checklist are classified in a numerical outcome (No = 0, Cannot Tell = 0.5 (when the provided information is insufficient or not clear), Yes = 1), with a maximum total score of 10. Following Butler et al. [17], we categorized the total CASP score for all studies in three categories (high, moderate, low) and the methodological quality was high (>8–10), moderate (6–8), or low (≤5). The third and last authors completed the CASP analysis independently and then discussed any disagreements before coming to an agreed consensus (kappa = 0.88). The overall methodological quality of the studies was good such that none of the articles had methodological problems that could affect the interpretation of our findings, which is consistent with narrative synthesis guidelines [18].

### 2.4. Data Extraction and Thematic Synthesis

In order to analyze the data, a qualitative methodology was used, as this is the method employed in the body of work on which this metasynthesis is based, as well as one of the articles’ inclusion criteria. The thematic synthesis was chosen as the qualitative evidence synthesis method for its usefulness to provide information [19]. The current study followed the approach to the synthesis of findings of qualitative research named “thematic synthesis” proposed by Thomas and Harden (2008) [20]. This method follows and is adhered to the Equator Network ENTREQ guidelines (Enhancing Transparency in Reporting the Synthesis of Qualitative Research [21]. The metasynthesis was started by reading each article multiple times and reflecting on the data, trying to respond to the first objective of the study and the research questions. All text under the headings ‘results’ or ‘findings’ was extracted electronically and entered with computer software package QSR NVivo (Version 12; QSR International Pty Ltd., Melbourne, Australia [22]), a software used for qualitative data analysis.

The synthesis followed three stages that were applied as follows:

#### 2.4.1. Stages 1 and 2: Coding Text and Developing Descriptive Themes or Sub-Themes

The study’s findings were extracted and summarized according to the research questions. As Thomas and Harden (2008) [20] suggest, and in order to avoid imposing a priori framework implied by the research questions onto study findings, they were temporarily put to one side. Thus, the process continued to develop from the study findings themselves to carry out a thematic analysis. They were entered into the NVivo software [22], and then each member of the research team independently coded each line of text based on its meaning and context. Free codes—without a hierarchical structure—were created inductively to capture the meaning and content of each sentence. Every sentence had at least one code applied, and the majority was categorized using several codes. This process created a total of 116 possible codes. The line-by-line coding allowed us to compare the concepts from one study to another, undertaking the translation process. At the same time, the synthesis process was started. Similarities and differences between the codes were identified, in order to group them into a structure. New codes were created to capture the meaning of groups of initial codes. This process resulted in a structure made up of ten subthemes.

#### 2.4.2. Stage 3. Generating Analytical or Main Themes

The descriptive themes that emerged from the inductive analysis of study findings were used to answer the research questions that had temporarily been put to one side: the family’s opinions about the supports received during the process of AAC implementation; the AAC characteristics that constitute barriers and facilitators to their use; and the challenges posed by the different contexts of use of the AAC. This process resulted in the generation of three main themes, from which different conclusions were extracted aiming to contribute to professional practice.

## 3. Results

### 3.1. Study Selection

The search yielded 1398 articles distributed over time as shown in Figure 1. After the removal of duplicates, 927 titles remained. Of these, 880 studies were excluded after the title and abstract screening, as they did not fulfill the eligibility criteria. Of the 47 studies that were full-text screened, 28 did not meet the inclusion/exclusion criteria. Specifically, 9 studies were excluded as they included adults (>18) in the sample of participants, 5 studies were excluded as parents’ perceptions were not reported, 13 studies were excluded as qualitative methodology was not used, and finally, 1 study was excluded as AAC systems were not employed.

### 3.2. Study Characteristics

A total of 19 studies were identified for inclusion in the current review as summarized in Table 1. All the included studies were scientific studies analyzing the parents’ perception or experiences of their children’s use of AAC.

The 19 studies summarized the perspectives of 297 parents, mainly mothers. The studies included 293 children and adolescents aged between 3 and 17 years old with diverse diagnoses: 67 children diagnosed with ASD, 39 with cerebral palsy, 15 with intellectual disability, 7 with Down syndrome, 123 with Angelman syndrome, 4 with Fragile X syndrome, 24 with language/communication disorders, 13 others (no specified diagnoses), and 1 study involved a mother of a child who was deaf/hard of hearing. The majority of studies came from the United States (n = 6, 31.6%), followed by Australia (n = 4, 21.1%), and Sweden (n = 2, 10.5%). There was one study from the United Kingdom, Canada, Ireland, Sri Lanka, Korea, Kenya, and Malaysia. Two of the studies from the United States involved Spanish- and Russian-speaking parents. Four studies were from monolingual families and the participants of the other studies were from a mixture of multilingual and ethnic backgrounds, although most investigations were unclear on this aspect.

As regards the modalities of AAC systems, participants used multimodal AAC systems according to Crowe et al.’s [8] classification: (i) unaided systems (natural gestures, manual signing); (ii) aided systems and low-technology (communication books, pictures, drawing boards, tangible objects); (iii) aided systems and high-technology (speech-generation devices or VOCAs, apps, mobile, iPads, computers) as well as The Picture Exchange Communication System (PECS). The design and methodology of the included studies were qualitative data derived from interviews (n = 15) or focus groups (n = 4). The most common methods of analysis were thematic analysis (n = 8) and narrative, descriptive, and content analysis (n = 5). Several investigations (n = 6) described the analytical process but did not specify the methodology of the analysis used. The sample sizes of children and adolescents of the 19 included studies were diverse, ranging from n = 4 to n = 122.

### 3.3. Thematic Synthesis

The inductive analysis of the different topics addressed in the selected studies led us to determine three broad themes and ten subthemes outlining parental experiences and perceptions of AAC for children with communication difficulties. The three main themes identified concerned service support, the characteristics of the AAC systems, and the integration of AAC into daily life. Inside each theme, different subthemes could be recognized. Regarding parents’ perceptions on service support, four subthemes were identified: parents’ support and training, accessibility, service providers, and coordination. With respect to the characteristics of AAC systems, parents’ contributions could be categorized into usability and acceptability, features, and cost and funding. Finally, issues related to the integration of AAC in daily life were organized into the different environments of AAC use: family, school, and social and community contexts. These key findings, organized into themes and subthemes, are synthesized in Table 2.

#### 3.3.1. Theme 1 Support

##### Subtheme 1: Parents’ Support and Training

Some studies showed that problems for supporting families were an important factor in AAC abandonment [23,24,26,27,31,34,41]. Parents expressed their desire of having a high level of support with a variety of service delivery models, such as telehealth, more parent-training courses or interventions, or family-to-family networking. Supplementary measures, such as online forums, AAC manuals, and websites were useful to some families but in general they required specific assistance and individualized support.

Most family members shared that it was challenging to find the ways to get the support they needed related to AAC:

*“I think that if we were really shown the full capabilities of the system by the speechie [SLP] then it would just be ingrained in what we’re doing every day.”*

[35] (p. 63)

*“We need to educate the teachers more… Because Anthony is not alone, and there are still [students] in this district who are completely nonverbal and don’t have a device.”…Until we got trained [on the SGD], it was like a foreign language. People say, “Oh you just have this machine.” But it’s really difficult unless you receive training … I don’t know, I just kind of wish we had more support, like maybe a person that can come in and make parents feel comfortable.”*

[27] (p. 270)

Consequently, parents expressed the need of individualized support, coaching, resources, and encouragement to use their child’s AAC system. However, this degree of support is difficult to achieve in many countries given logistical, resource, and policy barriers to service provision [37].

##### Subtheme 2: Accessibility

Parents indicated various settings in which AAC accessibility was difficult, for example when camping, when living in rural areas, in public places and noisy situations, or in the car:

*“I may not talk to her as much. You know what I mean? Cause with her brother I can talk to him in the car. But with (Child) because she can’t answer me in the car… I don’t ask her questions.”*

[24] (p. 242)

*“Nat, a parent who had previously lived in a rural area, remembered travelling long distances to see the specialist clinical team ‘every six months I think’.”*

[23] (p. 78)

Moreover, they mentioned some regulations that limited the use of AAC devices, as when insurance is not valid in another country.

##### Subtheme 3: Service Providers’ Support

Parents expressed their perceptions of service providers: SLPs, therapists, and other professionals [23,34,35,37,38,39]. In general parents advocated for better training and preparation of therapists before working with complex AAC systems.

*“The therapist’s expertise is insufficient”, “I think there’s a need for AAC education targeting special education teachers.”*

[37] (p. 325)

Similarly, families stated that the responsibility of teaching the child and establishing the device should initially fall to the SLP or therapist, with parents gradually implementing home teaching.

At the same time, families seemed to feel themselves devalued when professionals perceived themselves as the only ‘expert’ in AAC intervention:

*“They [the education department] treat you all like idiots, instead of collaborating, instead of sharing expertise they are just toxic and they couldn’t care less.”*

[34] (p. 52)

##### Subtheme 4: Service Coordination

Families also expressed their disappointment at the deficiency in cooperation among the institutions responsible for their children’s education and therapy or parental training:

*“We need more inter-institutional communication or arrangements.”*

[37] (p. 325)

Families were also concerned about overlapping between services within a team. Parents noted a lack of consistency in services for families with a new AAC system and called for improved communication and co-ordination of services within and across settings, organizations, professionals, and parents:

*“They’ve not received any supervision from their special educator. So it’s ignorance, and they should need more material too. And who’s responsible? But everyone is—so no one takes responsibility. It’s sad—though it was probably meant to be the other way around.”*

[41] (p. 376)

The overlap and miscommunication between therapists or services shown in some studies [23,31,39] could be minimized with clear communication and structured service planning, as some parents claimed:

*“Selena was diagnosed with cerebral palsy at 3 months, Charlie was swamped by the recommendations for home practice coming from Selena’s occupational therapist, physiotherapist, SLP, and others—at times adding to more than 27 hours of homework per day: ‘…and that didn’t take into account that she needed to be fed, she needed to sleep, she needed to have a bath, and she just needed to be a kid!’. As Charlie suggested: ‘I think if they started doing more of a multidisciplinary model… then everyone would be aware of what the other therapists were putting on the family…”*

[23] (p. 79)

#### 3.3.2. Theme 2 AAC Systems

##### Subtheme 5: Usability and Acceptability

Parents conveyed their perceptions about the usability and acceptance of the use of AAC by their children. Overall, the results were mixed [23,24,28,32,33,38,41]. Some families perceived AAC systems positively across parents from dissimilar socio-economic backgrounds. Specifically, parents perceived AAC (tablets, iPads, smartphones, PECS) as easy to use and portable, providing direct responses, being intuitive, and being patient and tireless interlocutors. Moreover, according to parents’ perceptions, the use of AAC systems facilitated not only communication development but also autonomy and self-esteem:

*“My daughter uses the iPad. She’s incredibly free to be like everyone else when she uses it, compared to many other things. Here she’s more on the same level as her brothers. Here, there are suddenly things that she’s able to do herself. Then it strengthens her self-esteem.”*

[41] (p. 375)

*“[PECS worked] pretty easily… he will just go up to them and he’ll tap them and he’ll show you what he wants.”*

[38] (p. 413)

Parents reported that these AAC tools were particularly useful in helping their children make simple requests: “*child does not have to work hard to get point across’ and knows what she is reaching for*.” [28] (p. 561)

In contrast, other families perceived concerns about the usability of the AAC devices, because of their experience of frequent device breakdowns. Parents expressed the importance of having fast and easy access to technical help: “*communication aids often had to be sent away for repair, sometimes leaving the children without a voice for several weeks, communication board or book that they used with direct access or partner-assisted scanning; these non-electronic forms of communication took more time and placed more demands on the communication partners*.” [24] (p. 242)

In addition, parents indicated that their children’s failures to understand or value the device could even lead to the rejection of the AAC systems involved. Likewise, parents reported not being familiar with the apps that were introduced to their child, thus experiencing difficulties using the app and the child employing it as a game more than communication: “*child doesn’t see benefit, ‘he viewed the device as a toy’ and he wanted to mouth the device rather than use it’ and thus being unmotivated to use their devices (e.g., ‘only interested in buttons of device’).”* [28] (p. 563)

##### Subtheme 6: Features

Parents provided insight on appealing design features for current and future AAC apps for their children [24,25,33,35,36,37,39]:

*“If it was customizable, and if there were 100 cartoon characters and you got to pick the one your child loves, that would be huge.”*

Parents also provided the idea that the character should “say the words” [25], p. 360, even raising the possibility of developing technology aimed at transforming thoughts into oral language:

*“I wish I could talk to Bill Gates and say ‘Hey, come on, come up with a program’ maybe something directly linked to the brain too, like the thought processing, when you think how advanced the technology is now, if they can have something linked to the brain to move limbs already, well why can’t they do that with the speech.”*

([24], p. 242)

In addition, some families expressed their concerns about the design, which might be visually confusing for their children. For example, the use of color-coding in the design was identified as an important component. Other parents viewed enhanced cursors as a potentially beneficial feature but indicated that this feature might be distracting for some children: “*My kids personally would concentrate more on the look of it, the different colors, than they would the actual communication choices*.” [25] (p. 563)

The physical design of technologies was another limitation. Parents also discussed limitations related to design functions and features. For example, limitations in technologies due to a lack of fit with their child’s needs. Thus, some parents discussed how their child’s system was organized alphabetically, even though he was not literate. Furthermore, the need for children to look at the screen of the AAC system may interfere with the kind of social orientation and visual contact required in daily communication. In this way, many informative cues conveyed through gaze and facial expression may be lost:

*“If she wants to communicate with the device she’s basically forced to look on a screen all the time and it’s a bit cold, you know. It’s not a good situation for someone who is as socially in tune as she is.”*

(Emily) [39] (p. 247)

Another parents’ perception was related to the need to mitigate the limited nature of the symbols, improve voice quality, and enhance the portability of AAC aids. They also highlighted that the characteristics of AAC systems entail a certain slowness to communicate through sentences:

*“It’s a burden to mothers. We have to look after our children while lugging the AAC aids around. The pictures are, shall I say, too hard. A style drawn in lovelier, softer lines, like our own traditional Korean style, seems like it would suit us more. The system is too simplistic. The process of forming sentences takes too long.”*

[37] (p. 325)

For low-technology systems, some parents expressed that the presence of too many small pictures, having to constantly prepare pictures, and pictures going missing when they were needed, was a problem. Moreover, parents hoped that high-technology systems, especially apps, will eventually be available in more local languages:

*“If she could use apps, at least there would be more choice, but now it seems like most apps are in English.”*

[33] (p. 116)

##### Subtheme 7: Cost and Funding

Some families expressed their problems and frustration to face the economic costs of the devices, as well as their maintenance. In this sense, parents mentioned facing financial challenges:

*“We got a quotation for a high-technology device, and it was very expensive. It is also difficult to find a sponsor.”*

[33] (p. 115)

The most frequently cited content that parents perceived to be valuable was to recognize the importance of AAC cost, including the provision of funding. Parents discussed their frustration regarding the lack of funding available for long-term support services, despite the substantial set-up costs incurred [23,26,33,35,36]:

*“I was beside myself, I was so angry that I’d been given this eleven and-a-half thousand dollar machine, and nobody in [the state] could give me any kind of support.”*

[23] (p. 78)

In some instances, parents did not perceive AAC systems as good value for money:

*“iPads are good but again there’s all that risk of the cost of them. So every time to fix a screen was $150 and after three times we just gave up and went well this is only gonna be in the house.”*

[36] (p. 5)

#### 3.3.3. Theme 3 Integration of AAC in Daily Life

##### Subtheme 8: Family

Most parents reported that they experienced difficulties finding time to support their child’s use of AAC and they felt that the use of AAC increases demands on their time. Families faced a multitude of challenges in daily family life, such as co-parenting, balancing the needs of siblings and family and employment commitments or lack of time to prepare materials. For example, they expressed their “difficulties sourcing pictures” for low-technology systems such as communication books and PECS. Therefore, parents felt frustrated because their responsibilities increased after the introduction of AAC [24,26,27,29,30,31,32,35,38,39,40]:

*“I work full-time as a nurse, and my husband works full-time, and three kids and two older kids who are running in six directions. And unfortunately, we probably fall short of making time [to support AAC use].”*

[27] (p. 270)

*“He wants to watch Sponge Bob all the time. Well he can’t, because there is 3 other kids. Or I am realistic so I know that I don’t work with her how I should I would like to, like sit with her and work, but then I started to think ok, you are working full time…”*

[29] (p. 204)

In sum, some parents described feeling overwhelmed because they had to deal with many responsibilities while seeking assistance and information of AAC services.

Furthermore, some parents preferred to rely primarily on unaided communication (gestures, partner-assisted scanning, or low-tech communication books) at home, especially in some daily life contexts as morning and bedtime care routines, when integrating AAC technologies (i.e., eye gaze devices) becomes a difficult challenge [39]. Moreover, parents are used to communicating with their children in a faster and more fluent way. This may reduce enthusiasm for AAC (i.e., food/drink PECS cards), particularly where it is seen to duplicate existing communication strategies.

*“I think he just comes back home and thinks I am off I don’t have to use them anymore, I have free time, don’t bother me with pictures.”*

[29] (p. 202)

##### Subtheme 9: School

Parents were most likely to talk about inclusive education as a facilitator, emphasizing the benefits on language and other learning outcomes [27,32,33,34,36,41]. They explained that a large part of the success in implementing an AAC system lies in the coordination and frequent contact with the teacher, which facilitates its widespread use:

*“Actually, there is a big improvement from using a Tablet I know this through experience. Now for my baby, I brought the Tab to the centre on Friday to get the app inserted… Now my baby started working on a Tab from last week. Now she sits and works on it for 1 1 = 2 hours. She is now interested in it. That’s because she is used to the things in it… Honestly, my baby was ill over the last 3 days. But even while she was ill I put her to work. That’s because I have got this into me. I feel that the child is getting something out of this. … I sent the teacher the work that she did everyday through Viber and WhatsApp. So they looked at what we did and its shortcomings and told us how to change things. So, daily, from Sunday to today, I video recorded the work and sent it through WhatsApp … When we have so much technology, there is no point if we don’t take advantage of it, no?”*

[32] (p. 188)

Other parents perceived the education system as not accessible or inclusive, as the use of the AAC was not promoted in the school:

*“At school they kept taking [the AAC system] off him. And so then he just started balking at it and would only use it for school work because that’s the rules. And so even at home he would, he stopped using it so except for his homework.”*

[35] (p. 63)

*“As long as I was quiet and didn’t understand my rights, I was a “good” parent. And it shouldn’t be like that … It’s all fine and dandy for a little while, but they stopped [providing inclusive services] and so I had to do a lot of advocacy, a lot of research to learn and things like that … There’s so many barriers, it’s so hard.”*

[27] (p. 270)

Additionally, some parents raised challenges associated with having a more restricted curricular focus and instructional tasks that weren’t viewed as being meaningful, limiting students with complex communications needs:

*“Evan, he hasn’t really been given a reading instruction… They don’t think children who are non-verbal can learn [to read]. So, they don’t really teach them.”*

[27] (p. 268)

##### Subtheme 10: Social and Community

Parents of younger and older children mentioned that their children spent most of their time with adults and were rarely left alone to interact with peers [26,27,40]. They recognized the importance of social interaction for their children and the need for a broader range of social opportunities after school and during leisure time. Communication was the preferred activity, although when people were not patient enough to wait for the child to produce the message, he or she would not participate fully in the communicative exchange and might become frustrated. Moreover, parents expressed their desire for their child to spend more time playing with peers instead of intensively working in communication abilities:

*“There were times—before the [name of communication device], she would want to be on the floor playing or whatever, but she will bypass playtime so she can communicate because that’s more important.”*

[24] (p. 244)

According to some parents, children with complex communication needs did not see peers after school or in shared activities, and few peers visited children at home because they did not want to communicate with them:

*“No-one comes. We’ve invited them but nobody comes. For her birthday this year it did work, two kids actually came. But we asked last year at the end of the school year if any of her classmates would mind just popping over in the summer time, spending an hour with her, or going to the movies with her, and no-one volunteered, so … [long pause] …”*

[24] (p. 245)

### 3.4. Methodological Quality of Included Studies

The methodological quality of all included studies was as follow: high (>8–10), moderate (6–8) or low (≤5) (see Table 3). The 10 questions (Q) corresponding to Program Qualitative Checklist (Critical Appraisal Skills Program (CASP) are listed below, and they were analyzed and filled out by the first and last author of our research team:

Q1. Was there a clear statement of the aims of the research?

Q2. Is a qualitative methodology appropriate?

Q3. Was the research design appropriate to address the aims of the research?

Q4. Was the recruitment strategy appropriate to the aims of the research?

Q5. Were the data collected in a way that addressed the research issue?

Q6. Has the relationship between researcher and participants been adequately considered?

Q7. Have ethical issues been taken into consideration?

Q8. Was the data analysis sufficiently rigorous?

Q9. Is there a clear statement of findings?

Q10. How valuable is the research?

Overall, the methodological quality of all included studies was deemed either high (n = 17) or moderate (n = 2) (see Table 3 for details). However, there were several issues that were identified. There were five studies (26%) in which there were doubts about the rigor of the data analysis reported and in five (26%), there were there were doubts as to whether the ethical issues had been considered. In fact, in two (10%) of these, there was no evidence these questions had been taken into consideration. In four studies (21%), the qualitative methodology was not clearly explained and in 3 studies (21%), it was not clear if the relationship between the researcher and the participants had been considered. One study (5%) did not report this question.

## 4. Discussion

This systematic literature review of 19 studies is the first comprehensive synthesis of the parental perceptions and experiences of their children use of AAC, using qualitative studies. A first purpose of the current review was to identify, integrate, and interpret common themes that may be relevant to extend current evidence-based practice. Findings resulted in the identification of three key themes in relation to service support. Moreover, this metasynthesis has significantly enhanced the very preliminary findings of Light and McNaughton [42], stressing that AAC technology should respond to the needs, skills, and preferences of children with complex communication needs and their families.

The three themes and subthemes provide novel insights into the parents’ perceptions about AAC service support, characteristics of AAC systems, and integration of AAC in daily life to develop a comprehensive understanding of the important concepts to be considered in the successful implementation of AAC systems. The first theme focused on service support. Parents perceived the need of a high and constant level of support as well as further training of therapists in AAC systems, in order to better understand their children’s demands. The parental perceptions’ outcomes are in line with previous findings in children with Autism Spectrum Disorder, emphasizing the utility of adapting service delivery models to different settings [12,13]. Moreover, changes in communication between service providers are necessary according to parental perceptions. Important aspects of communication with services providers (positive communication strategies) and delivery (home visiting and a collaborative, non-directive approach) and the value of constant support from professionals and their coordination (SLPs, medical services, teachers) were emphasized.

The second theme identified revolved around the characteristics of the AAC. Parents highlighted some aspects of the AAC design that make them more attractive and easier to learn for their children and the features that might lead to confusion. Some families emphasized the AAC ease of use and their contributions in their children’s communication and independence. However, other parents reported frequent technical problems that were not always quickly solved. An important consideration concerns the selection of the most appropriate AAC system for their children, which should fit their profile of strengths and needs. Another challenge that they perceived was the continuous adaptation of the AAC features to the needs of children with CCN. Added to this, parents mentioned other factors such as the children’s difficulties to understand the value of the device, as they perceived it as a toy, which may constitute a barrier to the learning process of the AAC and even to its abandonment.

Families also provided ideas for future technological advances that could enhance the communicative effectiveness of the AAC, for example, improving voice quality or the process of sentence formation and production. Furthermore, they underlined the high cost of many AAC devices and the demands for funding, especially for low-income families.

Finally, a third main topic was identified, which dealt with the parents’ experiences about the integration of AAC systems in their daily life. Parents conveyed many challenges: lack of time, care of other children, relationship between siblings, extended family, lack of AAC training, the need for a more inclusive educational system, and the limitations in social interaction presented by their children with complex communication needs.

A second objective of this study was to find out the quality of the research about family’s experiences and opinions on the use of AAC by their children. In this sense, it is important to underscore that the findings of this review are based on studies of high and moderate quality. Although this fact strengthens the soundness of the obtained conclusions, some aspects must be improved in future investigations, such as methodological rigor (e.g., research design, data analysis) or ethical issues.

Learning an augmentative and alternative communication system is a long process that requires a lot of effort, both for the children and for the significant people in their lives. Moreover, it is also often a difficult emotional journey in which families may feel lost and have many doubts about what is best for their children. This comprehensive review has revealed some significant issues raised by parents, which may provide guidance to professionals and other stakeholders to help them cope with this process. Parents should feel accompanied and empowered in the endeavor of equipping their child with a communication system. Although there is a great deal of information available in the form of manuals, websites, videos, and training, more individualized support is required in order to cover the multiple and continuous demands of children with severe communication disorders and their families. While recognizing the need of families’ empowerment, professionals must also be conscious of their busy schedules, and efforts should be made not to overburden them.

Families seem to advocate for transdisciplinary models of intervention composed of well-prepared professionals. This training and expertise must be extended to the school context, where teachers play a crucial role in generalizing the AAC use. A key related issue is the awareness of classmates about the functioning of AAC and their essential role in communicating with their peers with disabilities. This approach aligns with peer-mediated interventions designed to promote social interaction, a vulnerability area of children with complex communication needs. Additionally, parents have identified several environments where AAC accessibility is more difficult and, consequently, different actions must be carried out to break these barriers.

Finally, it is important to highlight that although AAC is a fundamental means of communication for many children with AAC, some parents prefer to rely primarily on natural modes of communication (facial expression, pointing, looking, body movements, etc.) as they sometimes seem to be faster, easier, and smoother than the use of an AAC system. Enhancing and validating that kind of natural communication is another way of optimizing the communicative potential of children with CCN and their families.

Summarizing, the results indicate that there are commonalities in challenges to AAC use within the included studies. These challenges consider different issues such as funding and time requirements, access to AAC tech, lack of skills, knowledge and training of service providers, lack of support from the AAC user’s team, and the impact of AAC in family, school, and social daily life. Additionally, challenges identified in the studies include the physical restrictions of the AAC user and the lack of adoption of AAC use in environments outside of the home.

### Limitations

This systematic review presents some limitations. Most notably, the review yielded only 19 studies, and most of them with a small number of participants and a relatively wide age range, limiting the ability to draw broader conclusions. Another limitation was the limited reporting of AAC typologies in the original studies. Knowing not just the type of AAC, but how it was introduced is important to understand how AAC impacts parents’ perceptions. Finally, the findings must also be considered with respect to methodological limitations of the few studies included.

Further research is needed that addresses the interactions between children with complex communication needs and their communication partners, which would help to conceptualize the nature of barriers and facilitators of using AAC systems in diverse environments. Similarly, research should aim to explore the interactions between children using AAC and their siblings or peers and identify siblings’ perceptions of AAC.

## 5. Conclusions

The present review investigates parents’ experiences and perceptions as regards their children’s use of AAC, and identifies barriers and key aspects related to the use AAC by children and adolescents with speech, language, and communication difficulties. To date, this is the largest and most comprehensive review of the qualitative literature of parents’ perceptions and experiences of AAC.

Findings suggest a need for more services that support children with complex communication deficits in different environments, more inclusive school programs (promoting meaningful engagement with peers), more functional use of AAC systems in real-world situations, and service support over an extended time period.

The current analysis has important implications for stakeholders (parents, SLPs, teachers, medical services, and community) in understanding AAC systems and providing support to children with communication disorders and their families.

## Figures and Tables

**Table 1:** Main characteristics of the studies (N = 19).

Author, Year, Country	Sample	Objective	Type of AAC	Study Design and Method	Main Themes
Anderson et al. [23], Australia	6 parents (2M, 4F) of 6 children (3M, 3F, 2–18 years old) with CP (n = 4), ASD (n = 2)	Perspectives and experiences of parents of children with an SGD	SGD	Qualitative study narrative analysis, semi-structured interview into themes and categories	Five primary themes were identified: (a) access to services, (b) therapist knowledge and expertise, (c) service continuity, (d) roles and responsibilities, and (e) parent power.
Batorowicz et al. [24], Canada	8 parents (2M, 6F) of 8 children (6F, 2M), 5–15 years old, IQ > 80, with communication production problems	Views of parents of children who use aided communication on social participation, communicative interactions, and relationships	Graphic communication system on a speech-generating device (SGD)	Qualitative study Thematic content analysis, semi-structured interview	Five themes were identified: (a) communication partners and strategies, (b) access to aided communication, (c) participation in society, (d) interaction opportunities, and (e) social relationships.
Boster et al. [25], USA	5 parents of 5 children with ASD (4–17 years old)	Parents’ views of children with ASD regarding appealing features of AAC applications	AAC app designs of: PECS, Sign Language, Vantage Lite, LAMP	Descriptive study Focus group interviews	Parents’ focus group provided insight on appealing design features for future AAC App: communicative mode, play mode, incentives.
Gona et al. [26], Kenia	10 Caregivers (5M, 5F) of 10 children (4CP, 4 ID, 1 Deafness, 1 ASD) 4–12 years old	Caregivers’ experiences about effects of a home-based intervention in AAC for children	home-based intervention using unaided AAC / PECS	Qualitative study semi-structured interviews	Four main themes emerged from the data: communication process; normality; struggle; and supernatural power.
Biggs et al. [27], USA	4 mothers of 2 children with ID, and 2 children with ASD (6–17 years old)	Perspectives of different stakeholders (parents) about challenges and facilitators to successful intervention for students who use AAC	Unaided AAC and Low-tech/High Tech	Qualitative study semi-structured interviews and content analysis	Findings revealed three areas across interacting ecological systems as being important determinants: AAC access, family–school partnerships and supports, and inclusive education.
Calculator et al. [28], USA	122 parents of children with AS (3–18 years old)	Parents’ views of using AAC including speech generating devices, in relation to other aided and unaided methods of communication	AAC devices VOCAs	Qualitative study thematic analysis, semi-structured interview	Reasons for children’s rejection and acceptance of the most advanced electronic AAC devices introduced to them within the past 3 years.
Doak [29], UK	5 mothers of 5 children with ASD (1F, 4M) 6–8 years old	Family’s perceptions of affordances and constraints of AAC used in the home	AAC applications (PECS cards, Makaton signing)	Qualitative study thematic analysis, semi-structured interview	Four themes identified: AAC in the family home, embodied idiosyncratic communication, competing household priorities, parents’ emotions.
Fäldt et al. [30], Sweden	16 parents of children 9M and 4F children (2–3 years old). 7 with ASD	Parents’ perceptions of AAC applications use and outcomes of their child’s communication	AAC applications during 1 year ComAlong Toddler Intervention	Qualitative study semi-structured telephone interviews	Four categories were identified: (a) Development for parents and the child, (b) acquiring useful tools, (c) useful learning strategies, and (d) benefits and challenges regarding intervention structure.
Glacken et al. [31], Ireland	18 parents (15F, 3M) of 18 children with DS, ASD, CP (2–11 years old)	Parents’ experiences of Lámh as a communication support to their child	AAC unaided system: Lámh, a key word signing approach	Qualitative exploratory research design thematic analysis, interviews	Three subthemes: Lámh potential, achieving and maintaining engagement (accessibility, training), speech and language therapy support, and the existence of a Lámh signing environment external to the home.
Hettiarachchi et al. [32], Sri Lanka/Ireland	16 parents (11F, 5M) of 16 children (9 CP, 3 ASD, 4 ID), 5–15 years old	Perceptions of parents in a resource-poor Global South country on the use of mobile technology as AAC devices	Multimodal AAC (mobile, smartphone, tablet, iPad, JABtalk app	Qualitative study focus group discussion, semi-structured interviews	Six broad themes emerged indicating a penchant for mobile technology, though its current use with their children was mainly as a teaching tool rather than a communication.
Singh, et al. [33], Malaysia	12 parents (10F, 2M), of 12 children (6 CP, 6 ASD) 3–12 years old	Malaysian parents’ perception of AAC and their experience	PECS, Makaton, communication book or board	Qualitative analysis, semi-structured interviews	Three main themes: (a) impact of the use of AAC, (b) challenges faced, and (c) hopes for the future.
Johnson et al. [34], Australia	9 parents of 9 children 2–17 years old with DS, ASD, ID	Parents perspectives of AAC in the self-directed funded service context of Kids Chat	Multimodal AAC service low-tech aids and apps	Qualitative study thematic analysis, semi-structured interview	The overall themes were accessing information, unrelenting responsibility, and looking to the future.
Moorcroft et al. [35], Australia	12 parents of 12 children (6F, 6M) 3–16 years old (5 ASD, 1 ID, 1 AS, 5 others	Parents perceptions on the contribution of external stakeholders to rejection or abandonment of an AAC system	Unaided and aided (low- and high-tech) AAC systems	Qualitative study Thematic analysis, semi-structured interview	Four themes: (1) parents were influenced by the beliefs of professionals; (2) parents did not feel supported by SLPs; (3) communication between stakeholders was not effective; (4) difficulties using AAC without a supportive community.
Moorcroft et al. [36], Australia	12 parents of 12 children (6F, 6M) 3–16 years old (5 ASD, 1 ID, 1 AS, 5 others	Parent perspectives on the contribution of factors associated with the family unit to the rejection or abandonment of an AAC	Unaided and aided (low- and high-tech) AAC systems	Qualitative study Thematic analysis, semi-structured interview	(a) Parents lacked resilience to implement AAC, (b) was extraneous work for parents, (c) the child did not use AAC, and (d) parents were not satisfied with AAC.
Park [37], Korea	12 mothers, 12 children (7M, 5F), 5–15 years old, 4 ASD, 4 CP, 1 ID, 1 language disorder, 2 others	Parents’ experiences with AAC	Low- and high-tech AAC systems: kids voices, tablet, PECS, communication boards/books	Qualitative study semi-structured interview	Seven themes related to parents’ experiences of AAC intervention, two themes regarding the factors affecting the acceptance of AAC.
Schladant et al. [38], USA	4 mothers of 4 children with FXS (4–12 years old)	Parents’ perspectives on AAC for children with FXS and how to improve communication outcomes	AAC System (PECS, communication boards)	Qualitative study semi-structured interview	Four themes identified: AAC usefulness, mothers attitudes, AAC experiences, stressors, need for support.
O’Neill et al. [39], USA	9 parents of 8 children with CP (6–14 years old)	Parent perspectives on how AAC technologies were integrated into everyday life	AAC technology (apps, iPad, SGD)	Qualitative study semi-structured interview	Themes: (a) integrating AAC into life, (b) AAC technologies, (c) child needs and skills, (d) parent responsibilities and priorities, and (e) AAC decision making.
Townsend et al. [40], USA	14 mothers of 14 children with ASD (4–17 years old)	African American mothers’ perceptions of the utilization of AAC by their children with ASD	AAC technology (aided and unaided)	Qualitative study semi-structured interview	Three themes were identified: AAC utilization, independence, and value of social interaction.
Wilder et al. [41], Sweden	15 parents (13F, 2M) of children with severe difficulties in communication (3–16 years old)	Understanding of how parents share learning about (AAC) for people with SD	Multimodal AAC, iPads, apps	Qualitative study focus group interviews, thematic analysis	Themes: AAC in School, AAC and Technology, AAC and holistic perspective. Communication form, Multimodal AAC is effective, New technology boosts the person’s confidence’, Cooperation among a person’s environments.

AAC: augmentative and alternative communication, ASD: Autism Spectrum Disorder; CP: cerebral palsy, M: male, F: female, SGD: speech generation device, ID: intellectual disability, AS: Angelman syndrome, DS: Down syndrome, SLPs: speech–language pathologists, FXS: Fragile X syndrome, SD: severe difficulties, PECS: Picture Exchange Communication System, VOCAs: voice output communication aids.

**Table 2:** Themes and subthemes.

Theme	Subtheme	Findings
	Parents’ support and training	Parents expressed their desire of having a high level of support with a variety of service delivery models and contexts
Service Support	Accessibility	Parents were concerned about accessibility difficulties in certain contexts
	Service providers’	Lack of therapist AAC knowledge and support for AAC appeared to be the biggest obstacle impacting AAC use
	Service coordination	Parents complained about the lack of communication between their service providers
Characteristics of AAC Systems	Usability and acceptability	Parents reported that these AAC tools were particularly useful, and they highlighted the importance of having fast and easy access to technical help
	Features	The technological characteristics of AAC systems should be adapted to the needs of families with children with CCN: more motivating features and a wide variety of resources and components
	Cost and funding	Lack of funding available for long-term support services
Integration of AAC in daily life	Family	Parents perceived that they must deal with many challenges: lack of time, care of other children, relationship between siblings, extended family, lack of AAC training
	School	Parents claimed for a more inclusive education system
	Social and community	Parents concerned about difficulties in their children’s social relationships

**Table 3:** Methodological quality assessment of included studies.

Reference	Q1	Q2	Q3	Q4	Q5	Q6	Q7	Q8	Q9	Q10	Total
Anderson et al. [23]	1	0.5	1	1	1	1	1	1	1	1	9.5 high
Batorowicz et al. [24]	1	1	1	1	1	1	1	1	1	1	10 high
Boster et al. [25]	1	1	0.5	1	1	1	1	1	1	1	9.5 high
Gona et al. [26]	1	1	1	1	1	1	1	0.5	1	1	9.5 high
Biggs et al. [27]	1	0.5	1	1	1	1	0.5	0.5	1	1	8.5 high
Calculator et al. [28]	1	0.5	1	1	1	1	1	0.5	1	1	9 high
Doak [29]	1	1	1	1	1	1	1	1	1	1	10 high
Fäldt et al. [30]	1	1	0.5	1	1	1	0	1	1	1	8.5 high
Glacken et al. [31]	1	1	1	1	1	1	1	1	1	1	10 high
Hettiarachchi et al. [32]	1	1	1	0.5	1	1	1	1	0.5	1	9 high
Singh, et al. [33]	1	1	1	1	1	1	1	1	1	1	10 high
Johnson et al. [34]	1	1	1	1	1	1	1	1	1	1	10 high
Moorcroft et al. [35]	1	1	1	1	1	0.5	0.5	1	1	1	9 high
Moorcroft et al. [36]	1	1	1	1	1	0.5	0.5	1	1	1	9 high
Park [37]	0.5	1	0.5	1	1	1	0	1	0.5	1	7.5 mod
Schladant et al. [38]	1	1	1	0.5	1	1	1	1	1	1	9.5 high
O’Neill et al. [39]	1	1	1	1	1	1	1	1	1	1	10 high
Townsend et al. [40]	1	1	1	0.5	1	0	1	0.5	0.5	1	7.5 mod
Wilder et al. [41]	1	0.5	1	1	1	0.5	1	0.5	1	1	8.5 high
% of Included studies rated as: ‘Yes’	94	78	84	84	100	78	73	73	84	100	
